# Anxiety, Depression and Posttraumatic Stress Disorder after Terrorist Attacks: A General Review of the Literature

**DOI:** 10.3390/bs11100140

**Published:** 2021-10-19

**Authors:** Claudia Rigutto, Adegboyega O. Sapara, Vincent I. O. Agyapong

**Affiliations:** Department of Psychiatry, Faculty of Medicine and Dentistry, University of Alberta, Edmonton, AB T6G 2R3, Canada; Adegboyega.Sapara@albertahealthservices.ca (A.O.S.); agyapong@ualberta.ca (V.I.O.A.)

**Keywords:** terrorism, anxiety, depression, post traumatic stress disorder

## Abstract

Terrorism, though not well-defined, is a violent act that has been shown to have longstanding effects on the mental health of those who witness it. The aim of this general literature review is to explore the effect that terrorism has on posttraumatic stress disorder (PTSD), major depressive disorder (MDD) and anxiety disorders, as well as the bio-psycho-social determinants that mediate its impact. This paper describes the prevalence, risk factors, protective factors, common presentations and interventions identified for PTSD, depression and anxiety disorders occurring following terrorist attacks. We conducted a literature search in MEDLINE using a number of keywords detailed below. After applying inclusion and exclusion criteria, we kept 80 articles, which we summarized in tabular form. A majority of articles found detailed the impact of terrorism on PTSD, and took place in a Western, mainly American setting. The main factors that impacted the presentation of mental illness include gender, ethnicity, social supports, socioeconomic status, level of preparedness, level of exposure, pre-existing trauma and mental illness, and subsequent life stressors. The main intervention detailed in this article as showing evidence post-terrorism is trauma-focused cognitive-behavioural therapy. This study highlights the importance of this topic, and in particular, its implications for public health policy and practice.

## 1. Introduction

There is no widely agreed upon definition of terrorism. In general terms, it is defined as an act of violence that is used to further a political goal by instilling fear into the public [1,2]. Though acts that could be defined as “terrorism” have occurred since the Roman Empire, the term was coined during the French Revolution [2]. Since the terrorist attack in New York City on 11 September 2001 (9/11), it is difficult to imagine a topic that has shaped global discourse as much as terrorism. In 2019, there were over 8300 terrorist attacks worldwide with about 25,000 fatalities from terrorism [2]. Over the course of the 21st century, fear of a terrorist attack occurring in proximity has grown, especially in the Western world. In 2018, over a third of individuals surveyed in countries such as Spain, Great Britain and Germany believed there would be an attack on home soil [3]. 

Academic interest in the association between terrorism and health, namely mental health, started in the late 90s. However, interest peaked following the 9/11 attacks. Most research centered around the newly christened diagnosis of posttraumatic stress disorder (PTSD) in the DSM-IV [4]. More than a decade later, research has also been done regarding the burden of other mental health conditions following a terrorist attack, such as major depressive disorder (MDD) and anxiety disorders; though to a lesser extent than PTSD [5]. Although there is an abundance of information and data on PTSD, anxiety disorders and MDD in other contexts, we are specifically curious regarding research in the context of terrorism. 

This article presents a review of the recent literature on the topic. In this paper, we aim to describe the prevalence, risk factors and protective factors related to the development of PTSD, anxiety disorders and MDD in the face of a terrorist attack, as well as discuss distinct symptom presentations in this context and any specific screening or other interventions that may be useful. Finally, conclusions and recommendations will be discussed.

## 2. Materials and Methods

This article explores and describes the most recent evidence on the impacts of terrorism on mental health, as well as social determinants and interventions that may mediate its effect. The search for articles was mainly completed by a single examiner, though there was a second examiner who aided in reviewing the admissibility of articles that did not fully meet the inclusion criteria. This review was conducted on MEDLINE (1946-present via Ovid); search terms included the following: 

“Terrorism”, “(terroris* or terror attack* or bioterrorism)”, “anxiety disorders/ or agoraphobia/ or anxiety, separation/ or neurotic disorders/ or obsessive compulsive disorder/ or hoarding disorder/ or panic disorder/ or phobic disorders/ or phobia, social/”, “((anxiety adj6 (disorder* or diagnos* or clinical* or illn*)) or obsessive compulsive disorder or OCD or panic disorder* or neurotic disorder* or hoarding disorder* specific phobia*)”, “depressive disorder/ or depressive disorder, major/ or depressive disorder, treatment-resistant/ or dysthymic disorder/”, “(depress* adj6 (disorder* or diagnos* or clinical* or illn* or major*))”, “stress disorders, post-traumatic/ or stress disorders, traumatic, acute/”, “(PTSD or PTSI or PTSS or ((posttraumatic or post traumatic or trauma*) adj1 (stress* or neurosis or neuroses or nightmare*)) or ((traumatic or acute) adj (stress disorder* or stress symptom*)) or (vicarious* adj2 trauma*))”. 

This search yielded 1632 articles. When narrowing the search to articles published between 2015 and the date of our search (December 2020), 436 articles were found. Removing duplicates yielded 390. We then reviewed titles, removing 229 unrelated articles, and yielding 161. Then, we reviewed the abstracts, removing 81 unrelated articles and keeping a total of 80 articles. Inclusion criteria was that the articles needed to assess an adult population and the aim needed to be related to risk/vulnerability factors, protective factors, presentation, screening or interventions related to PTSD, anxiety disorders or MDD specifically in the context of terrorism. We excluded case studies, qualitative articles, reviews examining child and adolescent populations, papers focusing on outcomes that were out of the scope of this research article (e.g., medical comorbidities), ethical, philosophical, legal or commentary papers, and analyses of the mental health of perpetrators of attacks. In total, 80 articles met all inclusion criteria. These were retained and summarized for the study purpose.

## 3. Results

The final 80 studies which met all inclusion criteria are summarized in Table 1, Table 2, Table 3, Table 4 and Table 5 below.

## 4. Discussion

### 4.1. Posttraumatic Stress Disorder

#### 4.1.1. Prevalence and Risk Factors

Post-traumatic stress disorder is an outcome that is commonly explored throughout research on terrorism. In the general American and European population, 1-year prevalence is between 0.9–3.5% [84]. The articles featured in this review identify a higher prevalence of PTSD in individuals both directly and indirectly exposed to terrorist attacks. Given the increase in terrorism research that occurred post 9/11, the majority of the articles featured in this review assessed those populations. In direct survivors, the prevalence of PTSD has been found to be around 30% [74]. It reaches approximately 39% in the first 6 months, and slowly decreases to about 22% after 1 year [74]. The prevalence of PTSD without any comorbidities in survivors was found to be about 4.1%, even 14–15 years after the attack [54]. In traditional relief workers, such as first responders and rescue workers, prevalence for PTSD is lower in the first 3 years, and slowly climbs up to 10%, peaking approximately 5–6 years post-attack [10,73]. In non-traditional relief workers, such as volunteer workers, rates of PTSD are much higher, climbing to 21.9% [10]. The prevalence is about 23% in relatives or close friends of victims who were injured or killed in terrorist attacks [74].

Though there is not an abundance of articles examining the mental health effects of terrorism set in countries other than the U.S.A., some of these have been reviewed for this article. Certain communities showed higher proportions of PTSD, others lower. However, it can be challenging to compare the prevalence rates given that the level of exposure of the populations studied is heterogenous. In Nairobi, the prevalence of PTSD in survivors and rescue workers following the 1998 U.S. Embassy bombing was 22% which was found to be 2–4 times the rates following the Oklahoma City bombings [26]. In the 4–6 weeks following the Bardo museum attack in Tunis in 2015, one study found that 68.6% of museum works displayed posttraumatic stress symptoms [13]. Similarly, 5 months following the Qissa Khwani Bazaar bombing in Pakistan, 77% of direct survivors suffered moderate to severe PTSD [6]. Following the 2015 Ankara bombings in Turkey, one study found that PTSD prevalence in direct survivors was 24.7% [12]. In contrast, following the 2011 Oslo bombing, only 2% of trained professionals and 15% of unaffiliated volunteers developed PTSD [24]. In the first 10 to 34 months, individuals who were directly exposed showed a prevalence of PTSD evolving from 24% to 17%, while for those who were indirectly exposed it went from 4% to 2% [71]. In France, following the November 2015 Paris terrorist attacks, prevalence amongst resident physicians was 12.4% [16] and between 3.4–9.5% in other first responders [44].

Pre-attack risk factors to developing PTSD include being a woman, being of Asian or Hispanic decent (in the American context), having been exposed to a previous terror attack, experiencing a traumatic event in childhood or adulthood, having low social and educational status and having pre-existing psychiatric comorbidities [8,13,15,22,28,33,40]. One study found that a genetic polymorphism of the serotonin transporter (5-HTT (5-hydroxy tryptamine)] gene) may have led to higher rates of PTSD post 9/11 [45]. Personality characteristics associated with PTSD include negative affectivity, detachment and psychoticism, as well as less perceived self-efficacy [51,54]. In first responders, having only basic life-saving training versus more intermediate or advanced training, was found to be a risk factor for PTSD [31]. During the terrorist attack, the main predictors for developing PTSD are level of exposure [48,73], including experiencing high perceived threat and having witnessed a life-threatening injury [12,36]. Higher perceived threat is a predictor for developing PTSD even in individuals who did not directly witness the attacks [36]. Following the terrorist attack, having low social supports, comorbid depression, anxiety and alcohol use have been shown to be risk factors for developing PTSD [8,13]. Suffering a physical injury secondary to the terrorist attack, regardless of the severity of the injury, is one of the biggest predictors of developing severe PTSD [22].

Regarding first responders, having had only basic life-saving training, as opposed to intermediate or advanced training, as well as having to intervene on unsecured crime scenes, likely leading to higher fear of death, were found to be risk factors for developing PTSD [31,44]. Certain studies also commented on risk factors associated with increased severity of PTSD. These include low social integration into the community, higher level of exposure to the attack, job loss following the event, marital status, unmet mental health needs, low education and socio-economic status, being a female and being of Hispanic descent [27,42,52,60]. In regard to symptomatology and comorbidities, risk factors for more severe PTSD include having severe hyperarousal symptoms, experiencing bereavement, being injured by the attack, having a history of PTSD, depression or anxiety pre-attack, having other medical conditions diagnosed post-attack, higher levels of exposure to the attack and a lifetime trauma burden, especially post attack [27,42,60]. Finally, from a temperament perspective, using coping strategies such as substance use and avoidance, as well as callousness and perceptual dysregulation personality traits, can worsen the trajectory of the illness [51,60].

#### 4.1.2. Protective Factors

When individuals and communities are exposed to terrorism, certain factors have been shown to protect against the development of mental illness. With regard to other forms of trauma, the general understanding is that adaptive coping strategies, greater social support and a sense of purpose are linked to lower PTSD symptoms [84]. These have similarly been shown to be protective factors in the context of terrorism exposure [60]. For first-line workers, feeling well prepared prior to the event, higher levels of training, feeling supported by leadership, lower role conflict, higher role clarity and predictability have shown to lead to lower rates of PTSD and less psychological distress [56,59,60]. More optimistic personality styles, benign styles of humour, perceived self-efficacy and the belief of having a life purpose all were traits that were associated with lower rates of psychological distress and post-traumatic symptomatology [54,55,57,60]. Individuals who employ problem solving and cognitive restructuring coping strategies were associated with fewer post-traumatic reactions and active coping skills distinguished between improving and chronic trajectories [60,62]. Finally, less severe emotional numbing symptoms was associated with higher rates of symptom recovery [27]. 

#### 4.1.3. Symptom Clusters and Course of Illnesses 

PTSD is often a chronic and highly disabling illness with an 18–50% recovery rate within the first 3–7 years. The four symptom clusters of PTSD include continuously reliving the traumatic event, persistent avoidance of stimuli related to the event, symptoms of emotional numbing and increased arousal response [84,85]. In first-line workers who intervened during terrorist attacks, especially volunteers, studies have shown that rates of PTSD continue to increase until a peak at 5–6 years post event. With regard to PTSD related to terrorism, even 6–7 years after the attack, 15–26% of direct victims continue to report PTSD symptoms [74]. Compared to other sources of PTSD, terrorism leads to a longer duration of illness (202 versus 92 months), with non-traditional workers showing the highest rates of chronic symptoms [67,75]. In one study, there was no statistically significant difference in the severity of symptoms between PTSD related to terrorism versus other forms of PTSD; however, higher avoidance symptoms were found, which is generally a severity marker [67]. However, one study out of Italy showed higher severity scores on the CAPS scale in terrorism than other forms of trauma [75]. When examining symptoms related to reliving the trauma, auditory reminders were the most frequently encountered and the most distressing [68,69]. One study found that the most central symptom seen in PTSD in the context of terrorism is feeling emotionally numb [66]. 

### 4.2. Major Depressive Disorder

#### Prevalence and Risk Factors

There are less studies available that look into the impacts of terrorism on rates of MDD. Rates of new-onset MDD post 9/11 were 26% amongst individuals who were in the vicinity of the attacks, and 45% amongst those with a trauma-exposed close associate [21]. Rates of MDD amongst community members and rescue workers alike were found to be 15.3% 10–15 years post attack [19]. Prevalence of MDD 12 years post attack was found to be 17.2% for non-traditional rescue workers and 30.3% for police officers [10]. Another study suggested that the majority of individuals directly exposed to terrorism, do not suffer from MDD in isolation. Even 14–15 years after the attack, 6.8% had MDD alone while 8.9% had comorbid MDD and PTSD [54]. When looking at individuals meeting criteria for PTSD following a terrorist attack, 68.2% also had comorbid MDD [54]. Another study showed similar numbers, with a prevalence of MDD 14–15 years post-attack being 18.6%, and over half those cases being associated with a diagnosis of PTSD [5]. Once again, prevalence can vary when looking into communities around the world. In Tunisia, following the Bardo Museum terrorist attacks, 40.6% of museum employees endorsed depressive symptoms after 4–6 weeks [13]. Conversely, only 2.4% of resident physicians reported depressive symptoms after the 2015 terrorist attack in Paris [16]. 

There are a number of factors identified in the literature which may lead to certain individuals having a higher likelihood of developing MDD after a terrorist attack. In general, for those who were directly exposed or who lived in the vicinity of a terrorist attack, being less educated, of lower socio-economic status, unemployed and having lower social integration and support increase their risk [5]. Those factors, along with traumatic life events post terrorist attack and chronic physical illness decreased the likelihood of recovering from MDD [5]. In a Tunisian study, low social support seemed to be the best predictor for both PTSD and MDD symptoms in directly exposed individuals [13]. Regarding chronic symptoms of MDD in the American context, less social support as well as less perceived self-efficacy were risk factors [54]. 

Regarding the level of exposure, some evidence suggests that individuals who lost a loved one to a terrorist act are more than twice as likely as direct witnesses to develop MDD [21]. In Norway, parents of victims of the attack were three times more likely to develop MDD and anxiety than the general population [50]. Furthermore, parental emotional reaction worsened the more symptomatic their child became [38]. MDD appears to be related to the magnitude of the attack’s impact on daily life, as well as how connected an individual is to the community affected [21]. In those who are not directly exposed to the attack, some evidence suggests that MDD can manifest only when an individual identifies the victims as being similar to their loved ones [37]. In those who were neither directly exposed nor had a loved one who was exposed, increased TV viewing related to the attack increased the likelihood of developing MDD, but only when it was associated with a decrease in perceived safety [32]. Similarly, in Nigeria, individuals who scored higher on the Terrorism Catastrophizing Scale were more likely to express symptoms of MDD and anxiety [7]. 

### 4.3. Anxiety Disorders 

#### Prevalence and Risk Factors

Individuals having survived a terrorist attack, especially those having suffered physical injuries, appear to report higher rates of anxiety disorders than the general population [25]. In the 10–11 years following the 9/11 attacks, 5.8% of police officers at the scene displayed comorbid anxiety and PTSD, and 47.7% had comorbid MDD and anxiety disorder [8]. Following the 13 November 2015 terrorist attacks which took place in Paris, 11.2% of resident physicians reported anxiety disorders [16]. In Denmark, rates of anxiety disorders saw a 16% increase following the Oslo bombings, and a 4% increase following 9/11 [17,18]. 

As with MDD, a multitude of factors can increase a person’s chances of developing an anxiety disorder following a terrorist attack. Some factors, such as scoring high on the Terrorism Catastrophizing Scale or having a loved one who was exposed to the attack overlap with the risk of developing MDD [7,50]. In Europe following an ISIS truck attack, physical proximity to the event, ISIS anxiety and perception of danger increased the risk for psychological distress in general [41]. Not only is physical proximity an important factor, one study found that cultural proximity also led to increases in trauma and stressor related disorders following terrorism. After the Oslo attack, the population of Denmark saw a spike in these disorders, independent to media coverage, possibly because of cultural and geographic proximity to the victims [18]. Regarding symptom severity, the frequency of exposures to reminders of the attack can lead to a worsening of MDD and anxiety disorders, as well as a global decline in functioning [69]. Those who endured traumatic experiences in their adulthood, as well as recent life stressors, were at higher risk of worrying about future terrorist attacks [33].

### 4.4. Interventions

Prior to beginning this section, it is important to note that this paper is not a review of all treatment modalities. Comments on interventions are based off of the treatment modalities detailed in the articles sampled. Following a terrorist attack, one study showed that over 25% of individuals with PTSD or MDD had unmet mental healthcare needs over the last year [19]. Individuals who were more likely to seek out counselling were Caucasians, Hispanics, children at the time of the attack, those with higher levels of exposure, those who experienced peri-event panic attacks and those who had accessed counseling prior to the attack [79]. Individuals who were least likely to seek out mental health support after 9/11 were found to be African Americans, Asian Americans, those of lower educational or economic status, and those without a regular physician [79]. Similarly, a study out of the U.K. highlighted that 2/3 of individuals exposed to terrorism who were connected to mental health supports, did so via their family physician [76]. Of those who accessed mental healthcare, individuals who rated counseling to be helpful were likely female, African American, over the age of 65 or those with very high exposure [79]. A study following the Utoya massacre noted that early outreach programs provided benefit to exposed individuals with and without PTSD, MDD and anxiety disorders [78]. However, it highlighted challenges in reaching certain populations, specifically individuals in modern family structures and ethnic minorities [78]. It is important to consider new and innovative means of connecting both the population at large and hard to reach populations to services and supports following trauma. One such means that has been recently described in the literature is supportive text messaging [86]. By providing personalized support to patients, mobile phone technologies have been found to potentially improve the outcomes of a number of mental health conditions such as MDD and possibly PTSD [86,87,88,89,90,91,92]. Similarly, a study from the U.K. highlighted the benefits in a general screening program for PTSD, MDD, anxiety disorders and alcohol use following a terrorist attack [76]. 

Trauma-focused cognitive-behavioural therapy (T-f CBT) has been found to be the therapy of choice for PTSD in victims of terrorism [77]. According to the articles sampled in this review, there is less evidence available regarding treatments of other mental disorders in victims of terrorism, as well as in non-developed, non-Western countries [77]. One study from Madrid examined a sample of survivors of terrorist attacks that occurred an average of 23 years prior. After a course of t-f CBT, prevalence of PTSD went from 23% to 3.2%, and anxiety disorders went from 14% to 9.7% [80]. Following the course of t-f CBT, no participants were expressing symptoms of MDD or panic attacks [80]. Significant decreases in symptoms were still present at a 1-year follow-up [80]. 

Improving one’s tendency to forgive and social supports, as well as working on other positive coping strategies, have been noted as possible therapeutic interventions following a terrorist attack, as these have been found to lower levels of PTSD symptoms [82]. Debriefing, and other crisis interventions in the first 24–72 h following terrorist attacks have been shown to lower quality of life and lead to worsening of MDD and PTSD symptoms [81,93]. According to the reviews sampled in this study, it was noted that there is limited information on treatment of mental illness following a terrorist attack. There is especially a lack of randomized–controlled trials in this area. However, there is some evidence highlighting the benefits of exposure-based approaches including virtual reality (VR) technology, as well as the use of SSRI medications [73].

## 5. Limitations

This literature review has some limitations. Firstly, the vast majority of articles studying the impacts of terrorism on mental illness occurred in developed countries, specifically the U.S.A; particularly in the post 9/11 context. This is the case even though 95% of deaths caused by terrorism occur in the Middle-East, Africa and South Asia [2]. Furthermore, articles in languages other than English were excluded, which further reduces the ability to assess studies published in other cultural contexts. Thus, it begs the question as to how culturally and globally representative the data presented in this review is. 

Secondly, this article consists of a general literature search, not a systematic review or scoping review. The authors did not use the PRISMA or PICO rules and triangulation method in the search for articles. It was mainly completed by a single examiner though there was a second reviewer who aided in reviewing the admissibility of articles that did not fully meet the inclusion and exclusion criteria previously agreed upon. In this study, only one database (MEDLINE) was used, which can introduce a bias towards medical sources and overlook non-medical, like psychological, literature. This method of reviewing the literature can introduce selection bias. As a result, it is presented as a qualitative summary of evidence found in a variety of articles. Notwithstanding the limitations of this review, it still provides an insightful overview of prevalence, risk factors, protective factors, symptomatology and possible management option of a mental illness in the context of terrorism. 

Lastly, it is important to note the lack of a widely agreed upon definition of terrorism. Indeed, the decision to deem one violent act as a terrorist attack versus another can often be subjective and may be racially biased. In general, there is a Western bias when defining events as terrorism or not, particularly in the post-9/11 climate. This bias is likely to have been reflected in the present study.

## 6. Conclusions and Future Directions 

It is challenging to identify a globally representative value for the prevalence of mental illness following a terrorist act, as the populations studied have been quite heterogeneous. In general, it appears that directly and highly exposed individuals with chronic physical sequelae following the incident, and underprepared (often volunteers) first-line workers, are populations with higher rates of PTSD. Populations that are geographically and culturally close to the victim population, as well as loved ones of victims, seem to have higher rates of MDD and anxiety disorders than the general population. Given that screening and outreach programs have been found to be effective in providing consistent mental health support to at risk populations, we wonder if on a systems level this would be needed to reduce negative outcomes following terrorism. 

As mentioned, there are many studies that examine the impacts of terrorism on mental disorders, specifically looking at prevalence and short-term illness progression. However, there are few studies that perform longitudinal assessments of illness trajectories in individuals exposed to terrorist acts. Studies of that nature would likely offer valid and valuable contributions to the field, especially with regard to the course of illness, and elucidating which factors and populations may be more at risk for developing chronic pictures versus resolution of symptoms. There would also be a benefit in further exploring the impact of terrorism on MDD and anxiety disorders, as there is at this point much more research looking into PTSD. 

In the studies sampled, it has been noted that, with regard to the study of treatments and interventions post-terrorism, there is limited information, especially with regard to a lack of randomized-control trials. Though there have been some interventions that have been validated in the management of terrorism-related mental illness, such as t-f CBT and SSRIs, more information is needed, especially in terms of long-term outcomes.

Though this was not a topic specifically covered by this study, considering risk factors for becoming a terrorist can be extremely clinically valuable in reducing harm. Given the tremendous impacts on mental illness that terrorist acts have on the general population, developing ways to detect someone’s risk of displaying terrorist behaviours is essential. Some papers have already compiled lists including social and clinical risk factors, and acts specific to terrorism that can be used as screening tools [94]. The UK’s counterterrorism strategy, CONTEST, which was established in 2003 is one such team that is looking into this. The Terrorism Radicalization Assessment Protocol (TRAP-18) has been identified as a tool that may be helpful for clinicians. Further research looking into risk and protective factors for becoming a terrorist may be useful [94]. 

It is important to note once more that this paper is a qualitative, general literature search as opposed to a systematic review or a scoping review. As mentioned, this can introduce significant bias in the information presented. As a result, we feel that future studies implementing scoping review or systematic review methods would be extremely useful to mitigate this bias. 

## Figures and Tables

**Table 1:** Prevalence of mental disorders after terrorist attacks.

Authors	Title	Methods	Key Findings
Abbas, S.A. et al., 2017 [6]	Impact of terrorism on the development of PTSD among the residents of Khyber Bazaar and its immediate surrounding areas in Peshawar, Khyber Pakhtunkhwa, Pakistan	Event, date & place: Pakistan, 9th October 2009 Type of study: cross-sectional survey, multicenter study N: 100 (survivors, rescuers and witnesses) Methods: structured interviews to assess PTSD severity 5 months post attack	66%: moderate PTSD 11%: severe PTSD Age, gender, occupation and education level did not have any correlation with PTSD development.
Abiola, T. et al., 2018 [7]	Fear of future terrorism: Associated psychiatric burden	Event, date & place: Kaduna bombings, Nigeria, April 2012 Type of study: cross-secrional survey N: 193 students and staff of Kaduna State University (KASU), Kaduna Polytechnic, and students awaiting admission into Kaduna State University Methods: sociodemographic questionnaire, the Terrorism Catastrophising Scale (TCS) and the depression and Generalised Anxiety Disorder (GAD) portion of the Mini Internation Neuropsychiatric Interview (MINI), 2 years post attack	78.8%: moderate to severe clinical distress related to fear of terrorism Moderate to severe clinical distress had a significant moderate correlation with depression.
Bowler, R.M. et al., 2016 [8]	Police Officers Who Responded to 9/11: Comorbidity of PTSD, Depression, and Anxiety 10–11 Years Later	Event, date & place: World Trade Center (WTC) attacks, 11th September 2001, New York City Type of study: cross-sectional study N: 1884 police officers enrolled in the WTC Health Registry Methods: participants categorized into four groups according to their level of comorbidity of PTSD, major depressive disorder and anxiety disorder. DSM-IV diagnostic criteria for PTSD used. Depression (PHQ-8) and anxiety (GAD-7) were assessed. Multinomial logistic regression was used to identify risk factors associated with comorbidity of PTSD.	12.9%: probable PTSD Of those, 21.8% had probable PTSD without comorbidity; 24.7% had major depressive disorder; 5.8% had anxiety disorder; 47.7% had comorbid major depressive disorder and anxiety disorder. Risk factors for comorbid PTSD, major depressive disorder, and anxiety disorder include being Hispanic, decrease in income, experiencing physical injury on 9/11, experiencing stressful/traumatic events since 9/11, and being unemployed/retired.
Bromet, E.J. et al., 2016 [9]	DSM-IV post-traumatic stress disorder among World Trade Center responders 11–13 years after the disaster of 11 September 2001 (9/11)	Event, date & place: World Trade Center (WTC) attacks, 11th September 2001, New York City Type of study: cross-sectional study N: 3231 responders who were monitored at the Stony Brook University World Trade Center Health Program Methods: 11–13 years post 9/11, master’s-level psychologists performed structured clinical interviews using the DSM-IV and the Range of Impaired Functioning Tool. The PTSD Checklist (PCL) and current medical symptoms were obtained.	9.7% had current, 7.9% remitted, and 5.9% partial PTSD. Avoidance and hyperarousal symptoms were most common, and flashbacks least common. PTSD was associated with health and well-being, mainly dissatisfaction with life.
Chen, C. et al., 2020 [10]	The Burden of Subthreshold Posttraumatic Stress Disorder in World Trade Center Responders in the Second Decade After 9/11	Event, date & place: World Trade Center (WTC) attacks, 11th September 2001, New York City Type of study: cross-sectional survey N: 4196 WTC responders (2029 police responders and 2167 non-traditional responders) registered in the WTC Health Program Methods: web-based surveys between 2012 and 2014, assessing demographics, WTC exposures, medical and psychiatric comorbidities, and mental health service use, and current probable PTSD level was assessed by using the PTSD Checklist.	Police offices: 9.3% had PTSD and 17.5% had subthreshold PTSD. Non-tradition: 21.9% had PTSD and 24.1% had subthreshold PTSD. Comorbid major depressive disorder was prevalent at 17.2% for non-traditional responders and 30.3% for police responders.
Cone J.E. et al., 2015 [11]	Chronic Probable PTSD in Police Responders in the World Trade Center Health Registry Ten to Eleven Years After 9/11	Event, date & place: World Trade Center (WTC) attacks, 11th September 2001, New York City Type of study: cross-sectional study N: 2394 police responders without pre-9/11 PTSD Methods: probable PTSD was assessed by the Posttraumatic Stress Check List (PCL). Risk factors for chronic, new onset or resolved PTSD were assessed using multinomial logistic regression.	50% of police with probable PTSD in 2003-2007 continued to have probable PTSD in 2011–2012. Risk factors for chronic PTSD included female gender, decreased social support, unemployment, life stressors in last 12 months, life-threatening events since 9/11, injuries during the 9/11 attacks, and unmet mental health needs
Essizoglu, A. et al., 2017 [12]	The risk factors of possible PTSD in individuals exposed to a suicide attack in Turkey	Event, date & place: suicide attack in Ankara, Turkey on 10 October 2015 Type of study: cross-sectional study N: 93 participants who had attended a meeting held in Ankara on that date Methods: completed a sociodemographic information form, the Traumatic Stress Symptom Checklist (TSSC), the Post Traumatic Cognitions Inventory, the Beck Depression Inventory (BDI), and the Influence of Perceived Societal Attitudes Questionnaire (IPSAQ)	24.7%: PTSD Risk factors for PTSD were being exposed previously to a suicide attack, witnessing a life-threatening injury, accessing psychological help, and having suicidal thoughts.
Fekih-Romdhane, F. et al., 2017 [13]	PTSD and Depression Among Museum Workers After the 18 March Bardo Museum Terrorist Attack	Event, date & place: the Bardo museum in Tunis, Tunisia, on 18th March 2015 Type of study: cross-sectional study N: 51 museum workers Methods: 4–6 weeks following the attack, PTSD and major depressive disorder were assessed using the Impact of Event Scale-Revised (IES-R) and DASS-depression scales. Information on sociodemographic factors and social support were gathered.	68.6%: PTSD 40.6%: major depressive disorder 35.3%: comorbid PTSD and major depressive disorder Risk factors for PTSD were low social support, advanced age and low eduction. Risk factors for major depressive disorder were low social support and advanced age.
Garfin, D.R. et al., 2018 [14]	Aftermath of Terror: A Nationwide Longitudinal Study of Posttraumatic Stress and Worry Across the Decade Following the 11 September 2001 Terrorist Attacks	Event, date & place: World Trade Center (WTC) attacks, 11th September 2001, New York City Type of study: longitudinal study N: 1613 U.S. residents Methods: beginning at the end of December 2006, used annual assessments administered every year for 3 years and assessed rates of 9/11-related posttraumatic stress (PTS) annually during the first 2 years of the study; during the second and third years of the study, assessed fear and worry regarding future terrorism.	Risk factor for PTS was both direct, and media-based (live television) exposure to the attacks 5–6 years later.
Goodwin, R. et al., 2017 [15]	Psychological distress and prejudice following terror attacks in France	Event, date & place: January 2015 Charlie Hebdo attack (1981) and the November 2015 Bataclan concert hall/restaurant attacks (1978), in France Type of study: panel survey N: 1981 (first attack) and 1878 (second attack) French citizens Methods: 4 weeks after the attacks, they measured intrinsic religiosity, social and traditional media use, psychological distress (K6), probable posttraumatic stress symptoms (proposed ICD-11), symbolic racism and willingness to interact with Muslims by non-Muslims.	Rates of probable PTSD were 11.9 and 14.1% for the two attacks.
Gregory, J. et al., 2019 [16]	The impact of the Paris terrorist attacks on the mental health of resident physicians	Event, date & place: 13 November, 2015, terrorist attacks took place in Paris Type of study: cross-sectional study N: 680 Parisian resident physicians Methods: anonymous questionnaires, including the IES-R and the Hospital Anxiety and Depression Scale (HADS), were emailed two months after the attacks. Exposure to the attacks was defined as having direct clinical contact with one of the victims up to one week after the attacks, being one of the victims, or having one among close relatives.	12.4%: PTSD 11.2%: anxiety disorders 2.4%: major depressive disorder
Hansen, B.T. et al., 2016 [17]	Increased Incidence Rate of Trauma- and Stressor-Related Disorders in Denmark After the 11 September 2001, Terrorist Attacks in the United States	Event, date & place: World Trade Center (WTC) attacks, 11th September 2001, New York City Type of study: longitudinal study N: 1,448,250 contacts with psychiatric services in the Kingdom of Denmark Methods: they utilized population data from the Danish Psychiatric Central Research Register (1995–2012), and performed a time-series intervention approach to estimate the change in incidence rate of mental disorders in the Kingdom of Denmark after the 9/11 attack.	16% increase in the incidence rate of trauma and stressor related disorders which lasted up to 1 year post attack. The impact of terrorist attacks on mental health is likely not limited to inhabitants of the target country.
Hansen, B.T. et al., 2017 [18]	Increased Incidence Rate of Trauma- and Stressor-related Disorders in Denmark After the Breivik Attacks in Norway	Event, date & place: 22nd July 2011, Norway, shooting and children in Norway Type of study: longitudinal study N: 159,618 acute contacts with Danish psychiatric hospital services Methods: using population-based data from the Danish Psychiatric Central Research Register (1995–2012) they examined a change in incidence in trauma related disorders after these attacks.	Geographic proximity to terrorist attacks is associated with increased psychological burden. Media attention may lead to a subsequent surge in incidence and substantial negative psychological reactions.
Jacobson, M.H. et al., 2018 [5]	Longitudinal determinants of depression among World Trade Center Health Registry enrollees, 14–15 years after the 9/11 attacks	Event, date & place: World Trade Center (WTC) attacks, 11th September 2001, New York City Type of study: longitudinal study N: 21,258 enrollees of the World Trade Center Health Registry Methods: completed four questionnaires over 14 years of follow-up. PTSD status was measured using the PTSD checklist and depression was assessed using the Patient Health Questionnaire (PHQ).	18.6%: major depressive disorder Risk factors for major depressive disorder were low income, unemployment, low social integration and support, post-9/11 traumatic life events, chronic physical illness and previous PTSD.
Jordan, H.T. et al., 2019 [19]	Persistent mental and physical health impact of exposure to the 11 September 2001 World Trade Center (WTC) terrorist attacks	Event, date & place: World Trade Center (WTC) attacks, 11th September 2001, New York City Type of study: longitudinal study N: 36,897 participants in the WTC Health Registry Methods: completed baseline (2003–2004) and follow up (2015–2016) questionnaires. Physical sequelae were obtained from surveys. PTSD was assessed using the PTSD checklist, and depression, the PHQ. Poor health related quality of life was defined as reporting limited usual daily activities for >14 days during the month preceding the survey.	14.2%: PTSD 15.3%: major depressive disorder Over 25% of participants with PTSD or major depressive disorder reported unmet need for mental health care in the preceding year. Quality of life was particularly low among participants with mental health conditions.
Kung, W.W. et al., 2018 [20]	Posttraumatic stress disorder in the short and medium term following the World Trade center attack among Asian Americans	Event, date & place: World Trade Center (WTC) attacks, 11th September 2001, New York City Type of study: longitudinal study N: 2431 Asian Americans and 31,455 non-Hispanic Whites from the World Trade Center (WTC) Registry Methods: 2–3 years and 5–6 years after the attack, participents divided into four categories PTSD pattern groups: resilient, remitted, delayed onset, and chronic. Multimodal regression analyses were performed.	Asians had chronic and remitting presentations of PTSD than caucasians. Risk factors for chronic and delayed onset PTSD in both races were immigrant status, disaster exposure, lower income, pre-attack depression/anxiety, and lower respiratory symptoms.
North, C.S. et al., 2015 [21]	The postdisaster prevalence of major depression relative to PTSD in survivors of the 9/11 attacks on the world trade center selected from affected workplaces	Event, date & place: World Trade Center (WTC) attacks, 11th September 2001, New York City Type of study: cross-sectional study N: 373 employees of 9/11-affected New York City workplaces Methods: completed a full diagnostic assessment methods categorization of exposure groups based on DSM-IV-TR criteria for PTSD.	26%: major depressive disorder 14%: PTSD Risk factors for major depressive disorder were the magnitude of the disaster impact, the personal connectedness of community members, having a trauma-exposed close associate and losing a loved one to the event.
North, C.S. et al., 2018 [22]	A Study of Selected Ethnic Affiliations in the Development of Post-traumatic Stress Disorder and Other Psychopathology After a Terrorist Bombing in Nairobi, Kenya	Event, date & place: 1998 terrorist bombing of the US Embassy in Nairobi, Kenya Type of study: cross-sectional study N: 392 exposed individuals, members of 7 major ethnic groups Methods: 8 to 10 months after the disaster, participants were assessed during a diagnostic interview using the DMS-IV. Demographic characteristics, disaster exposures and injuries, and ethnic affiliations was gathered.	PTSD was less prevalent in Kikuyu group. MDD was more prevalent in the Meru group. Risk factors for PTSD were past psychiatric history, disaster injuries.
Skogstad, L. et al., 2015 [23]	Exposure and posttraumatic stress symptoms among first responders working in proximity to the terror sites in Norway on 22 July 2011—a cross-sectional study	Event, date & place: 2011 Norway attack Type of study: cross-sectional study N: 238 various first-line workers Methods: a questionnaire was completed, 8–11 months after the attack looking into the prevalence and predictors of PTSS using the PTSD Checklist (PCL-S).	1.3%: PTSD Dissociative symptoms were found to be important predictors of developing PTSD. The scores were not significantly different amongst the various professions.
Skogstad, L. et al., 2016 [24]	Post-traumatic stress among rescue workers after terror attacks in Norway	Event, date & place: 2011 Norway terror attacks Type of study: cross-sectional study N: 1790 general and psychosocial health care personnel, police officers, firefighters, affiliated and unaffiliated volunteers Methods: questionnaires were conducted 10 months after the attack and participants were assessed for PTSD using the PCL-S.	0.3%: PTSD among trained personel 15%: PTSD among unaffiliated volunteers Risk factors for PTSD were being of female gender, witnessing injured/dead, perceived threat, perceived obstruction in rescue work, lower degree of previous training and being unaffiliated volunteers.
Tucker, P. et al., 2016 [25]	Intensely Exposed Oklahoma City Terrorism Survivors: Long-term Mental Health and Health Needs and Posttraumatic Growth	Event, date & place: Oklahoma City bombings, 1995 Type of study: cross-sectional study N: 407 directly exposed Methods: 18 and ½ years post-attack, telephone surveys compared terrorism survivors and non-exposed community control subjects, using Hopkins Symptom Checklist, Breslau’s PTSD screen, Posttraumatic Growth Inventory, and Health Status Questionnaire.	23.2%: PTSD
Zhang, G. et al., 2016 [26]	Psychiatric disorders after terrorist bombings among rescue workers and bombing survivors in Nairobi and rescue workers in Oklahoma City	Event, date & place: the 1998 U.S. Embassy bombing in Nairobi and the 1995 Oklahoma City bombing Type of study: comparative study N: 52 male rescue workers (Nairobi), and 105 directly exposed male civilian survivors (Nairobi) and 176 male rescue workers (Oklahoma) Methods: the Diagnostic and Statistical Manual of Mental Disorders-IV was used to assess pre-disaster and post-disaster psychiatric disorders, and variables related to demographics, exposure, disaster perception and coping was gathered.	22%: PTSD in Nairobi rescue workers and civilians 27%: MDD in Nairobi rescue workers and civilians The prevalence of PTSD and major depressive disorder after the Nairobi attack was 2–4 times higher than among rescue workers in Oklahoma City.

**Table 2:** Risk factors for development of mental disorders after terrorist attacks.

Authors	Title	Methods	Key Findings
Adams, S.W. et al., 2019 [27]	Posttraumatic Stress Trajectories in World Trade Center Tower Survivors: Hyperarousal and Emotional Numbing Predict Symptom Change	Event, date & place: World Trade Center (WTC) attacks, 11th September 2001, New York City Type of study: longitudinal study N: 2355 World Trade Center tower survivors Methods: surveyed using the WTC Health Registry data at about 2.5, 5.5 and 10.5 years following the attack to evaluate PTSD trajectories using latent growth mixture modeling. Sociodemographic characteristics, WTC-related exposure, and other traumas/stressors were assessed.	The majority of participants had stable symptom trajectory over time. Factors associated with worsening symptoms were being male, post World Trade Center life stressors and severe hyperarousal symptoms (particularly anxious arousal). Factors associated with symptom recovery were having less severe emotional numbing symptoms.
Bowler, R.M. et al., 2017 [28]	Posttraumatic Stress Disorder, Gender, and Risk Factors: World Trade Center Tower Survivors 10 to 11 Years After the 11th September 2001 Attacks	Event, date & place: World Trade Center (WTC) attacks, 11th September 2001, New York City Type of study: comparative study N: of 1755 World Trade Center evacuees Methods: 10–11 years after the 9/11 attacks, factors associated with PTSD symptom severity were examined using the PTSD Checklist.	Predictors of PTSD symptom severity were being younger when the attack occurred, unemployed, less educated, higher exposure to attack, unmet mental health care needs, less social support.
Bugge, I. et al., 2015 [29]	Physical injury and posttraumatic stress reactions. A study of the survivors of the 2011 shooting massacre on Utøya Island, Norway	Event, date & place: 2011 Utoya attacks Type of study: longitudinal study N: 325 survivors Methods: interviewed 4–5 months and 14–15 months following the attacks to assess physical injury, PTSD using the UCLA PTSD-RI scale, peritraumatic exposure, sociodemographic and psychosocial backgrounds.	Physical injury following the attack was associated with higher rated of PTSD, regardless of the severity of the injury
Cozza, S.J. et al., 2019 [30]	Patterns of Comorbidity Among Bereaved Family Members 14 Years after the 11th September 2001, Terrorist Attacks	Event, date & place: World Trade Center (WTC) attacks, 11th September 2001, New York City Type of study: cross-sectional study N: 454 bereaved family members Methods: separated them participants 3 groups: healthy, comorbid without PTSD and comorbid with PTSD. The contribution of non-9/11 lifetime traumas, pre-9/11 mental health conditions, post-9/11 interim life events, grief services, income adequacy, and social support was assessed.	Having comorbid psychiatric conditions increased the risk of developing PTSD, major depressive disorder and anxiety disorders.
De Stefano, C. et al., 2018 [31]	Early psychological impact of Paris terrorist attacks on healthcare emergency staff: A cross-sectional study	Event, date & place: attacks in Paris on 13 November 2015 Type of study: cross-sectional N: 130 healthcare workers who participated directly in the rescue efforts Methods: questionnaires less than 1 month following the attacks in which PTSD symptoms were measured by the Trauma Screening Questionnaire (TSQ).	Risk factors for PTSD were direct participation in rescue efforts, female gender and only receiving basic life-saving training (versus intermediate or advanced). The most reported symptom from both groups was hypervigilance.
Fullerton, C. et al., 2019 [32]	Active Shooter and Terrorist Event-Related Posttraumatic Stress and Depression: Television Viewing and Perceived Safety	Event, date & place: October 2002 Washington DC sniper attacks Type of study: cross-sectional study N: 1238 Washington, DC area residents Methods: assessed using an internet survey including the IES-R, the PHQ, hours of TV, and perceived safety.	TV viewing and decreased perceived safety were related to increased PTSD and major depressive disorder.
Garfin, D.R. et al., 2020 [33]	Exposure to Prior Negative Life Events and Responses to the Boston Marathon Bombings	Event, date & place: the 2013 Boston Marathon bombings Type of study: longitudinal study N: 846 individuals from Boston, 941 from New York and 2888 from the rest of the United States who had reported exposure Methods: assessed for prior negative life events and PTSD symptoms as defined by the DSM-V, ongoing fear about future terrorism and functioning.	Negative life events were related to higher PTSD. Childhood and adulthood trauma, and recent stressors were associated with poorer functioning.
Gargano, L.M. et al., 2016 [34]	Mental Health Status of World Trade Center Tower Survivors Compared to Other Survivors a Decade After the 11 September 2001 Terrorist Attacks	Event, date & place: World Trade Center (WTC) attacks, 11th September 2001, New York City Type of study: comparative study N: 7695 adult civilians that were part of the World Trade Centre Health Registry (1946 evacuees and 5749 nearby) Methods: logistic regression looking into PTSD and binge drinking, 10 years post attack.	Increased exposure and fear for life were risk factors to developing PTSD.
Gargano, L.M. et al., 2017 [35]	Resilience to post-traumatic stress among World Trade Center survivors: A mixed-methods study	Event, date & place: World Trade Center (WTC) attacks, September 11th 2001, New York City Type of study: mixed-methods study N: 7695 adult civilians that were part of the World Trade Centre Health Registry (1946 evacuees and 5749 nearby) Methods: logistic regression looking into PTSD and binge drinking, 10 years post attack.	Survivors had increased PTSD versus witnesses.
Heir, T. et al., 2016 [36]	Thinking that one’s life was in danger: perceived life threat in individuals directly or indirectly exposed to terror	Event, date & place: the 2011 Oslo bombing Type of study: cross-sectional study N: 1970 exposed individuals Methods: survey done 10 months following the attack assessed perceived life threat using by asking the question: “How great do you think the danger was that you would die?” and scoring on a five-point scale. PTSD was measured with the PCL.	High perceived threat was associated with PTSD among those directly and indirectly exposed.
Herberman Mash, H.B. et al., 2016 [37]	Identification With Terrorist Victims of the Washington, DC Sniper Attacks: Posttraumatic Stress and Depression	Event, date & place: 2002 Washington, SC sniper attack Type of study: cross-sectional study N: 1238 residents of the area Methods: 3 weeks following the attacks, participants completed the IES-R and PHQ-9 scales, and answered questions pertaining to identification with victims of the attacked. Three tyes of identification with victims were described: as like oneself, as like a friend, and as like a family member. PTSD and major depressive disorder post-terrorist attack.	PTSD prevalence was higher when identifying the with victims as themself or a loved one. Major depressive disorder prevalence was only higher when the individual identified with the victims as loved ones.
Holt, T. et al., 2017 [38]	Emotional reactions in parents of the youth who experienced the Utøya shooting on 22 July 2011; results from a cohort study	Event, date & place: Utøya shooting on 22 July 2011 Type of study: open cohort study N: 531 parents of youth who had been exposed to attackMethods: with assessments at 2 points: 4–5 months and 14–15 months post attack, outcomes were assessed using the Parental Emotional Reaction Questionnaire, and University of California, Los Angeles Post-traumatic Stress Disorder Reaction Index.	Mothers experienced more emotional distress than fathers. Parental emotional reactions worsened the more there was post-traumatic stress reactions in offspring, regardless of the age of the child.
Jose, R. et al., 2018 [39]	Mapping the Mental Health of Residents After the 2013 Boston Marathon Bombings	Event, date & place: 2013 Boston Marathon Bombings Type of study: longitudinal study N: 788 Boston metropolitan area residents Methods: spatial patterning of acute stress score, PTSD and fear was done and Data was collected 2–4 weeks to 2 years after the attack.	Higher distress was found in individuals in closer proximity to the attack sites.
Kung, W.W. et al., 2018 [40]	Factors Related to the Probable PTSD after the 9/11 World Trade Center Attack among Asian Americans	Event, date & place: World Trade Center (WTC) attacks, 11th September 2001, New York City Type of study: cross-sectional, comparative study N: 4721 Asian Americans was compared to 42,862 non-Hispanic White Americans Methods: 2–3 years following 9/11, data on prevalence, risk and protective factors for PTSD was collected.	There were higher rates of PTSD among Asians Americans compared to Caucasians. Higher education was a risk factor for Asian Americans, as opposed to Caucasians. Risk factors for PTSD were being an immigrant, level of exposure to the disaster and the presence of lower respiratory symptoms.
Mahat-Shamira, M. et al., 2018 [41]	Truck attack: Fear of ISIS and reminder of truck attacks in Europe as associated with psychological distress and PTSD symptoms	Event, date & place: ISIS truck attacks in Europe Type of study: cross-sectional study N: 397 adults living in Europe Methods: surveyed online 72 h after an ISIS terror attack, completed a PHQ scale and answered questions regarding their physical proximity to the event, associative memory of prior events, danger perception and ISIS anxiety.	Risk factors for psychological distress including major depressive disorder and anxiety disorders were physical proximity to the attack, ISIS anxiety, perceived danger of terror attacks, being reminded of the truck attack in Berlin.
Maslow, C.B. et al., 2015 [42]	Trajectories of Scores on a Screening Instrument for PTSD Among World Trade center Rescue, Recovery, and Clean-Up Workers	Event, date & place: World Trade Center (WTC) attacks, 11th September 2001, New York City Type of study: longitudinal study N: 16,488 rescue and recovery workers Methods: over 8–9 years, they assessed severity of PTSD using the PTSD Checklist.	Risk factors for developing more severe symptomatology include level of exposure (including duration of exposure), being injured from the attack, level of perceived threat to life or safety, bereavement, lower social support, marital status and unemployment
Monfort, E. et al., 2017 [43]	Traumatic stress symptoms after the 13th November 2015 Terrorist Attacks among Young Adults: The relation to media and emotion regulation	Event, date & place: Paris 2015 terrorist attacks Type of study: cross-sectional study N: 451 young adults who were not directly exposed Methods: trauma history, traumatic symptoms, media consumption, psychological distress and emotion regulation strategies were assessed via an online survey.	In individuals who were indirectly exposed, social network use and dysfunctional emotion regulation strategies were found to be risk factors for increased emotional distress.
Motreff, Y. et al., 2020 [44]	Factors associated with PTSD and partial PTSD among first responders following the Paris terror attacks in November 2015	Event, date & place: Paris November 2015 Type of study: cross-sectional study N: 663 first responders Methods: a web-based interview was performed 12 months following the attack and PTSD was assessed using the DSM-V criteria.	Prevalence of PTSD: 3.4% in firefighters, 9.5% in police officers Risk factors of PTSD were low educational level, social isolation, lack of training and intervention on unsecured crime scenes.
Olsson, A. et al., 2015 [45]	Neural and genetic markers of vulnerability to post-traumatic stress symptoms among survivors of the World Trade Center attacks	Event, date & place: World Trade Center (WTC) attacks, 11th September 2001, New York City Type of study: retrospective study N: 17 highly exposed survivors Methods: the relationship between PTSD symptomatology, a common genetic polymorphism of the serotonin transporter and neural activity response was assessed using functional MRI while showing images associated with 9/11 and images not associated as a control.	Carriers of the short allele had higher levels of PTSD. Posterior cingulate cortices activity mediated the relationship between the genotype and PTSD.
Pfefferbaum, B. et al., 2020 [46]	Media Contact and Posttraumatic Stress in Employees of New York City Area Businesses after the 11 September Attacks	Event, date & place: World Trade center (WTC) attacks, 11th September 2001, New York City Type of study: comparative study N: 254 employees of New York City businesses (105 exposed and 149 unexposed) Methods: 35 months after the attacks, structured interviews and questionnaires were administered to assess the relationship between PTSD and media contact.	Media coverage had no impact on psychopathological outcomes, but worsened re-experiencing and hyperarousal symptoms.
Pfefferbaum, B. et al., 2016 [47]	Reactions of Oklahoma City bombing survivors to media coverage of the 11 September, 2001, attacks	Event, date & place: 1995 Oklahoma City bombing Type of study: comparative study N: 99 survivors and 61 unexposed Oklahoma City community residents Methods: 2–7 months following the 9/11 attacks were assessed for emotional reactions and media behaviour.	Television viewing was related to post-9/11 PTSD. Surviving prior attacks worsened reactions to subsequent attacks.
Schwarzer, R. et al., 2016 [48]	A PTSD symptoms trajectory mediates between exposure levels and emotional support in police responders to 9/11: a growth curve analysis	Event, date & place: World Trade Center (WTC) attacks, 11th September 2001, New York City Type of study: longitudinal study N: 2204 exposed police officers Methods: assessed using the PCL scale and rated their received emotional support. Exposure levels were assessed via a yes or no questionnaire with possible scores ranged from 0 to 5.	Higher levels of initial trauma exposure led to worse long-term PTSD symptom severity.
Sugiyama, A. et al., 2020 [49]	The Tokyo subway sarin attack has long-term effects on survivors: A 10-year study started 5 years after the terrorist incident.	Event, date & place: the 1995 Tokyo subway sarin attack Type of study: longitudinal study N: 747 survivors Methods: using self-rating questionnaires collected annually by the Recovery Support Center between 2000–2009, the prevalence of PTSD using the IES-R scale was assessed over 10 years. The multivariate Poisson regression model was then used to estimate the risk ratios of age, gender, and year factor on the prevalence of PTSD.	35.1%: PTSD Risk factors for developing PTSD were old age and female.
Thoresen, S. et al., 2016 [50]	Parents of terror victims. A longitudinal study of parental mental health following the 2011 terrorist attack on Utøya Island	Event, date & place: 2011 terrorist attack on Utøya Island Type of study: longitudinal study N: 531 mothers and fathers of exposed children Methods: assessed at 5 and 14–15 months following the Utoya shooting, their rates of PTSD, anxiety disorders and major depressive disorder were compared to age and gender adjusted expected scores of the same illness in the general population.	Parents of exposed children had 3 times higher rates of major depressive disorder and anxiety disorders than the general population, and rates of PTSD were 5 times higher.
Waszczuk, M.A. et al., 2018 [51]	Maladaptive Personality Traits and 10-Year Course of Psychiatric and Medical Symptoms and Functional Impairment Following Trauma	Event, date & place: World Trade Center (WTC) attacks, 11th September 2001, New York City Type of study: longitudinal study N: 532 WTC responders Methods: completed the personality inventory for DSM-V. Their mental and physical health was assessed annually in the following decade.	Personality domains associated with PTSD were negative affectivity, detachment and psychoticism. Personality traits associated with worse PTSD illness trajectory for PTSD were callousness and perceptual dysregulation.
Welch, A.E. et al., 2016 [52]	Trajectories of PTSD Among Lower Manhattan Residents and Area Workers Following the 2001 World Trade Center Disaster, 2003–2012	Event, date & place: World Trade Center (WTC) attacks, 11th September 2001, New York City Type of study: longitudinal cohort study N: 17,062 adults who were both residents and workers (nonrescue and recovery) in the area of the WTC, included in the WTC Health Registry Methods: they administered the PTSD Checklist on 3 occasions over a period of 9 years. They examined factors associated with improving or worsening PTSD symptoms.	48.9% of participants had low severity and a stable trajectory of PTSD. Risk factors for worsening PTSD symptoms included low social integration, higher terror exposure, job loss and unmet mental health needs
Wesemann, U. et al., 2018 [53]	Burdens on emergency responders after a terrorist attack in Berlin	Event, date & place: 19th December, 2016 attack in Berlin Type of study: pilot study N: 37 (16 firefighters, 6 police officers, 5 psychosocial health care personnel and 9 members of aid organizations) Methods: 3 months following the attack, they were assessed using PHQ-9, The World Health Organization Quality of Life (WHOQOL-BREF), PCL-5 and the Brief Symptom Inventory (BSI) scales.	Females scored higher in stress and paranoid ideation. Police officers scored higher in hostility. Firefighters scored lower in quality of life, environment and physical health.

**Table 3:** Preventative and vulnerability factors against development of mental disorders after terrorist attacks.

Authors	Title	Methods	Key Findings
Adams, S.W. et al., 2019 [54]	PTSD and Comorbid Depression: Social Support and Self-Efficacy in World Trade Center Tower Survivors 14–15 Years After 9/11	Event, date & place: World Trade Center (WTC) attacks, 11th September 2001, New York City Type of study: longitudinal study N: 1304 survivors Methods: examined the association between mental illnesses, namely PTSD and major depressive disorder, and possible protective factors including social support and self-efficacy by assessing at 4 points: 2003–2004, 2006–2007, 2011–2012 and 2015–2016.	4.1%: PTSD alone 6.8%: major depressive disorder alone 8.9%: comorbid PTSD and major depressive disorder Risk factors for comorbid PTSD and MDD included greater exposure to the events of 9/11 and lower self-efficacy. Less perceived social support predicted only major depressive disorder and not PTSD, whereas less perceived self-efficacy equally predicted both.
Besser, A. et al., 2015 [55]	Humor and Trauma-Related Psychopathology Among Survivors of Terror Attacks and Their Spouses	Event, date & place: terror attacks in Israel Type of study: cross-sectional study N: 105 dyads consisting of Israelis who were injured during terror attacks and their spouses Methods: examined the associations between the use of different styles of humour and trauma-related psychopathology.	Protective factors for developing PTSD, MDD or anxiety disorders included benign styles of humor, the use of self-enhancing humor and the use of affiliative humor.
Birkeland, M.S. et al., 2015 [56]	Associations between Work Environment and Psychological Distress after a Workplace Terror Attack: The Importance of Role Expectations, Predictability and Leader Support	Event, date & place: the 2011 Oslo bombing attack Type of study: cross-sectional study N: 1800 ministerial employees Methods: 10 months after the attack, examined the impact of work environmental factors on levels of psychological distress following a workplace terrorist attack.	Lower role conflicts, higher role clarity, higher predictability, and higher leader support were independently associated with lower psychological distress.
Birkeland, M.S. et al., 2017 [57]	Does Optimism Act as a Buffer Against Posttraumatic Stress Over Time? A Longitudinal Study of the Protective Role of Optimism After the 2011 Oslo Bombing	Event, date & place: the 2011 Oslo bombing at the Norwegian gouvernment Type of study: longitudinal study N: 256 ministerial employees Methods: assessed using survey data collected 1, 2 and 3 years following the attack. They examined the relationship between optimism and development of PTSD and its specific symptoms clusters (intrusions, avoidance, numbing, dysphoric arousal and anxious arousal).	Optimism may help to neutralize the effects of high exposure on levels of symptoms of avoidance, numbing, and dysphoric arousal but not on the symptoms of intrusions and anxious arousal
Birkeland, M.S. et al., 2017 [58]	For Whom Does Time Heal Wounds? Individual Differences in Stability and Change in Posttraumatic Stress After the 2011 Oslo Bombing	Event, date & place: the 2011 Oslo bombing at the Norwegian gouvernment Type of study: prospective study N: 256 ministerial employees Methods: surveyed 10, 22 and 34 months following the attack.	Risk factors for PTSD include high exposure, female sex, and high levels of neuroticism. High exposure lead to higher levels of PTSD Social support was associated with lower severity of PTSD.
Dale, M.T.G. et al., 2020 [59]	Post-traumatic stress reactions and doctor-certified sick leave after a workplace terrorist attack: Norwegian cohort study	Event, date & place: the 2011 Oslo bombing at the Norwegian gouvernment Type of study: prospective cohort study N: 94 Norwegian governmental employees who met criteria for PTSD using the PCL-S Methods: questionnaire gathering data on the psychosocial work environment 10 months following the attack, and sick leave in the 12–22 months period following the attack.	Employees with PTSD after workplace terrorism would benefit from control over their workplace conditions and increased predictability to reduce the risk of sick leave.
Feder, A. et al., 2016 [60]	Risk, coping and PTSD symptom trajectories in World Trade Center responders	Event, date & place: World Trade Center (WTC) attacks, 11th September 2001, New York City Type of study: longitudinal study N: 4487 responders (traditional and non-traditional responders) Methods: assessed an average of 3, 6, 8, and 12 years post-9/11. Symptoms were categorized into different trajectory types and demographic and other information was gathered.	Protective factors for PTSD found were perceived higher life purpose, perceived preparedness, positive emotion-focused coping, active coping, greater perceived social support and availability of social support. Risk factors for worse PTSD symptomatic trajectories include being Hispanic, female, low educational status, low economic status, history of PTSD depression or anxiety pre-9/11, other medical conditions diagnosed post-9/11, level of exposure to 9/11, lifetime trauma burden (especially post 9/11), coping via substances, and avoidance coping strategies
Hem, C. et al., 2016 [61]	Effort-Reward Imbalance and Post-Traumatic Stress After a Workplace Terror Attack	Event, date & place: the 2011 Norway attack Type of study: cross-sectional study N: 1927 ministry employees Methods: data was collected 10 months after the attack, information on effort and any reward received was gathered, and its impact on PTSD were examined.	Employees who reported extra effort displayed increased risk for PTSD rates. Perceived reward for extra effort from a leader was associated with lower risk for PTSD.
Jensen, T.K. et al., 2015 [62]	Coping responses in the midst of terror: The July 22 terror attack at Utoya Island in Norway	Event, date & place: 22nd July 2011 terror attack on Utøya Island Type of study: cross-sectional study N: 325 survivors Methods: interviewed face-to-face 4–5 months after the shooting to examine peri-trauma coping responses and their relation to subsequent PTSD. The “How I Cope Under Pressure Scale” (HICUPS) was administered and PTSD was assessed using the UCLA PTSD reaction index.	Problem solving and positive cognitive restructuring were associated with fewer symptoms of PTSD.
Richardson, K.M., 2015 [63]	Meaning reconstruction in the face of terror: An examination of recovery and posttraumatic growth among victims of the 9/11 World Trade Center attacks	Event, date & place: World Trade Center (WTC) attacks, 11th September 2001, New York City Type of study: qualitative study N: 118 directly exposed individuals selected from a group of volunteer docents at the Tribute World Trade Center Visitor Center Methods: surveyed about their experiences.	The ability to make sense of one’s experience and the ability to find some benefit in the experience were protective factors.
Rosen, R. et al., 2019 [64]	Longitudinal Change of PTSD Symptoms in Community Members after the World Trade center Destruction	Event, date & place: World Trade Center (WTC) attacks, 11th September 2001, New York City Type of study: longitudinal study N: 738 individuals from the enrolled in the WTC Environmental Health Center at Bellevue Hospital Methods: identified patient characteristics associated with probably PTSD and longitudinal PTSD symptoms using the PTSD Checklist.	Risk factors for PTSD include being Hispanic, low economic status, below high school education, lower respiratory symptoms, physical symptoms, comorbid depression, comorbid anxiety and alcohol use. Risk factors for persistent PTSD included race, ethnicity and premorbid depression.
Tucker, P. et al., 2018 [65]	Do Direct Survivors of Terrorism Remaining in the Disaster Community Show Better Long-Term Outcome than Survivors Who Relocate?	Event, date & place: 1995 Oklahoma City Bombings Type of study: comparative study N: 138 survivors (114 still living in Oklahoma City, 24 having left) Methods: assessed using a telephone survey 19 years following the attack. Assessed their levels of PTSD, anxiety disorders, MDD, life satisfaction, posttraumatic growth, medical conditions, alcohol and cigarette use.	There was no statistical difference in the prevalence of mental illness between the two groups.

**Table 4:** Symptom clusters and illness trajectories.

Authors	Title	Methods	Key Findings
Birkeland, M.S. et al., 2017 [66]	Making connections: exploring the centrality of posttraumatic stress symptoms and covariates after a terrorist attack	Event, date & place: the 2011 Oslo bombing Type of study: cross-sectional study N: 190 survivors Methods: surveyed 10 months after the event with the aim of identifying the most central symptoms of posttraumatic stress, and to explore how factors such as exposure, sex, neuroticism, and social support are related to the network of symptoms of posttraumatic stress.	The following connections were identified: intrusive thoughts and nightmares, feeling easily startled and overly alert, feeling detached and emotionally numb, female and high physiological reactivity to reminders. Most central symptom: feeling emotionally numb
Bossini, L. et al., 2016 [67]	PTSD in victims of terroristic attacks—a comparison with the impact of other traumatic events on patients’ lives	Event, date & place: no specific event studied Type of study: comparative study N: 84 subjects suffering from PTSD who had been referred to a clinic in Italy between 2003 and 2014 (42 were terrorist attack survivors, and 42 had experienced other traumatic events) Methods: assessed using clinical interview, CAPS scale and DTS scale.	The duration of illness was longer in the terrorism group. There was no statistically significant difference regarding severity of symptoms between the groups. Terrorism leads to higher avoidance symptoms, while other traumatic events lead to higher hypervigilance symptoms. Avoidance has been found to be a severity marker and delays treatment access.
Durodie, B. et al., 2019 [4]	Terrorism and post-traumatic stress disorder: a historical review	Event, date & place: no specific event Type of study: historical review N: 400 research articles, for the most part published after 11 September 2001 Methods: examined the association between terrorism and mental health.	Terrorism may not cause greater than expected frequency of PTSD when compared to other traumatic events.
Glad, K. A. et al., 2016 [68]	Posttraumatic stress disorder and exposure to trauma reminders after a terrorist attack	Event, date & place: the 2011 Utoya Island attack Type of study: cross-sectional study N: 285 survivors Methods: interviewed 14–15 months after the event. They were asked to qualitatively and quantitatively define their symptomatology. PTSD was assessed using the University of California at Los Angeles PTSD Reaction Index.	Auditory reminders were most frequently encountered. most distressing and highly associated with meeting criteria for PTSD. 6.3%: PTSD
Glad, K.A. et al., 2017 [69]	A Longitudinal Study of Psychological Distress and Exposure to Trauma Reminders After Terrorism	Event, date & place: the 2011 Utoya Island attack Type of study: longitudinal study N: 261 survivors Methods: were interviewed face-to-face 14–15 and 30–32 months post event. They were asked to quantify and qualify their trauma reminders. Their level of PTSD was assessed using the University of California at Los Angeles PTSD Reaction Index and the Hopkins Symptom Checklist.	Higher frequency of exposure to trauma reminders lead to higher severity of PTSD, anxiety disorders, and major depressive disorder. 20% continued to be very distressed by reminders even 2.5 years post-event.
Hamwey, M.K., 2020 [70]	Post-Traumatic Stress Disorder among Survivors of the 11 September, 2001 World Trade Center Attacks: A Review of the Literature	Event, date & place: World Trade Center (WTC) attacks, 11th September 2001, New York City Type of study: literature review N: 30 articles included Methods: articles selected for inclusion were peer-reviewed empirical articles published in English from 2002–2019 that had collected data from directly exposed adults, examining information on prevalence and risk factors of PTSD and its comorbidities.	PTSD prevalence and course over time are determined by exposure to the events of 9/11, including the degree of exposure and the number of exposures. Persistent PTSD is predicted by 9/11 exposure severity and 9/11-related job loss. 50% of individuals with PTSD had comorbid major depressive disorder.
Hansen, M.B. et al., 2017 [71]	Prevalence and Course of Symptom-Defined PTSD in Individuals Directly or Indirectly Exposed to Terror: A Longitudinal Study	Event, date & place: the 2011 bombing in the government district of Oslo Type of study: longitudinal study N: 3520 employees Methods: survey data collected 10, 22, and 34 months after the attack. Prevalence of PTSD was assessed using the PTSD Checklist.	2–4%: PTSD with indirect exposure 17–24%: PTSD with direct exposure
Horn, S.R. et al., 2016 [72]	Latent typologies of posttraumatic stress disorder in World Trade Center responders	Event, date & place: World Trade Center (WTC) attacks, 11th September 2001, New York City Type of study: cross-sectional study N: 4352 WTC responders Methods: DSM-IV PTSD criteria used, latent class analyses identified a 3 PTSD typologies: high-symptom, dysphoric and threat.	Life stressors and premorbid mental illness leads to high symptoms presentation. Individuals in the high symptoms category most frequently screened positively for depression and functional impairment.
Lowell, A. et al., 2018 [73]	9/11-related PTSD among highly exposed populations: a systematic review 15 years after the attack	Event, date & place: World Trade Center (WTC) attacks, 11th September 2001, New York City Type of study: systematic review N: 20 longitudinal studies including 13 prevalence studies and 7 treatment studies Methods: peer-reviewed reports published between October 2001 to May 2016 with the aim of assessing prevalence and long-term trajectories of PTSD in individuals who were highly exposed to the World Trade Center terrorist attacks.	Rates of PTSD generally decline over time. PTSD trajectory: lower prevalence in the first 3 years, then there is a substantial increase to an estimated prevalence rate of 10%, and a possible peak at 5–6 years Risk factors for PTSD were exposure intensity (primary risk factor), 9/11 related injury and job loss (related to chronicity of PTSD symptoms), constant reminders of the trauma, such as physical pain, may be a comorbid expression of the disorder and/or help maintain it Treatments proposed were exposure-based approaches including using virtual reality technology, SSRI medications.
Paz Garcia-Vera, M. et al., 2016 [74]	A Systematic Review of the Literature on Posttraumatic Stress Disorder in Victims of Terrorist Attacks	Event, date & place: no specific event Type of study: systematic review N: 35 electronic and hand searched studies Methods: articles on PTSD among victims of terrorist attacks which included studies addressing the prevalence of PTSD using validated diagnostic interviews.	29.8%: PTSD in survivors, decreases after 6–12 months 15–26%: PTSD after 6–7 years 6.9%: PTSD in emergency, rescue, assistance, or recovery personnel or volunteers 23%: PTSD in relatives and close friends of the victims injured or killed, decreases after 6–12 months Risk factors for developing PTSD were level and length of exposure, level of destruction and death, professional training vs. volunteers. Among bereaved relatives and close friends: 18.5% had comorbid PTSD, major depressive disorder, and complicated bereavement; 8.6% had comorbid PTSD and major depressive disorder; 5.7% had PTSD and complicated bereavement; 5.7% had only PTSD
Pozza, A. et al., 2019 [75]	The Effects of Terrorist Attacks on Symptom Clusters of PTSD: a Comparison with Victims of Other Traumatic Events	Event, date & place: no specific event Type of study: comparative study N: 108 patients who had been referred to an Italian hospital (44 had been exposed to terrorism and 64 to other traumatic events) Methods: examined PTSD symptom clusters according to the traumatic event type by administering the Clinician-Administered PTSD Scale (CAPS).	Longer duration of untreated illness (202 vs. 92 months) and higher symptom severity were seen in the terrorism group.

**Table 5:** Screening tools and interventions.

Authors	Title	Methods	Key Findings
Cyhlarova, E. et al., 2020 [76]	Responding to the mental health consequences of the 2015–2016 terrorist attacks in Tunisia, Paris and Brussels: implementation and treatment experiences in the United Kingdom	Event, date & place: terror attacks in Tunisia, Paris and Brussels 2015–2016 Type of study: comparative study N: 529 individuals who had been offered screening by the Screen and Treat Program in the UK (75 of survivors) Methods: explored efficacy in identifying and referring people to mental health services, examining the programme’s acceptability to users and understanding how agencies involved worked together.	Almost all found it unhelpful regarding treatment of PTSD.
Garcia-Vera, M.P. et al., 2015 [77]	Efficacy and clinical utility (effectiveness) of treatments for adult victims of terrorist attacks: A systematic review	Event, date & place: no specific event Type of study: systematic review N: 8 studies, all on PTSD, 7 of which examined trauma-focused cognitive-behavioral therapy and 1 examined exposure therapy in combination with a serotonin reuptake inhibitor. Methods: examined the efficacy and clinical utility of treatments for mental disorders in adult victims of terrorism.	Trauma-focused cognitive behavioral therapy is efficacious and useful in clinical practice for the treatment of PTSD in victims of terrorism.
Haga, J.M. et al., 2015 [78]	Early postdisaster health outreach to modern families: a cross-sectional study	Event, date & place: the 2011 Utoya massacre Type of study: cross-sectional study N: 453 survivors Methods: survey with face-to-face interviews and questionnaires were done 4–7 months following the attack. Early outreach programmes were launched in all municipalities affected, and engagement with the team, utilisation of healthcare services and mental distress using the UCLA PTSD-RI and HSCL-25 scales were used.	Early outreach programmes showed good engagement in individuals with and without PTSD, major depressive disorder and anxiety disorders. There were challenges reaching individuals from modern family structures and ethnic minorities.
Jacobson, M.H. et al., 2019 [79]	Characterizing Mental Health Treatment Utilization among Individuals Exposed to the 2001 World Trade Center Terrorist Attacks 14–15 Years Post-Disaster	Event, date & place: World Trade Center (WTC) attacks, 11th September 2001, New York City Type of study: longitudinal cohort study N: 35,629 enrollees of the WTC Health Registry Methods: data up to 15 years post-disaster was used to examine predictors of counseling after 9/11, the types of practitioners seen, and the perceived helpfulness of therapy up to 15 years post-disaster.	Individuals who sought counselling the soonest: White and Hispanic Americans, those who were children during 9/11, those who sought counselling previously and those with high levels of exposure to the attacks. Least likely to seek counselling: Black and Asian Americans, those with lower education and income, males and youth.
Moreno, M. et al., 2019 [80]	Effectiveness of trauma-focused cognitive behavioral therapy for terrorism victims with very long-term emotional disorders	Event, date & place: no specific event Type of study: prospective study N: 50 victims of terrorist attacks who presented isolated or concurrent PTSD, MDD, panic disorder, or other anxiety disorders Methods: a course of trauma-focused cognitive behavioral therapy was administered average of 23 years post attack. An intention to treat analysis was performed to assess its efficacy.	At post-treatment and at the 1, 3, 6 months, and 1 year follow-ups, large statistically and clinically significant decreases in PTSD, MDD and anxiety disorders were found using trauma-focused cognitive behavioral therapy.
Tran, D.V. et al., 2018 [81]	The Association Between Dissatisfaction with Debriefing and Post-Traumatic Stress Disorder (PTSD) in Rescue and Recovery Workers for the Oklahoma City Bombing	Event, date & place: the 1995 Oklahoma City bombings Type of study: retrospective study N: 166 firefighters Methods: structured diagnostic interview assessing for PTSD using the DSM-III-R, and surveys regarding their level of satisfaction with debriefing.	Being “very dissatisfied” with debriefing was associated with symptoms of avoidance and numbing, as well as higher rated of PTSD.
Weinberg, M., 2018 [82]	The Mediating Role of Posttraumatic Stress Disorder with Tendency to Forgive, Social Support, and Psychosocial Functioning of Terror Survivors	Event, date & place: no specific event, Israel Type of study: cross-sectional study N: 108 terror survivors who suffered from a disability caused by the terror attack recognized by Israel’s National Insurance Institute Methods: examined the relationship between psychosocial functioning and tendency to forgive, social support and PTSD symptoms by administering the Psychosocial Adjustment to Illness Scale, the PTSD Symptom Scale–Self-Report, the Heartland Forgiveness Scale, the Multidimensional Scale of Perceived Social Support and a demographic questionnaire.	Higher levels of tendency to forgive and social support were associated with lower levels of PTSD symptoms Social support has both a direct and indirect effect on psychosocial functioning in individuals with PTSD following a terrorist attack.
Wesemann, U. et al., 2020 [83]	Impact of Crisis Intervention on the Mental Health Status of Emergency Responders Following the Berlin Terrorist Attack in 2016	Event, date & place: the Berlin Terrorist Attack in 2016 Type of study: comparative study N: 55 directly exposed emergency responders (37 underwent debriefing, 18 did not) Methods: assessed outcomes between the two groups using the PHQ-9, WHOQOL-BREF, PCL and BSI.	Lower quality of life and increased major depressive disorder were found in the group who had undergone crisis intervention.

## Data Availability

This review article is not associated with any primary or secondary data.
